# Primary prevention of Parkinson’s disease: proceedings, the 8C’s and a position statement from the Parkinson’s disease prevention think tank

**DOI:** 10.1038/s41531-026-01437-1

**Published:** 2026-06-23

**Authors:** Sneha Mantri, Rhoda Au, Anastasia Barnes, James C. Beck, Kathleen Blake, Peggy Borum, Wendy Dahl, Briana de Miranda, E. Ray Dorsey, Ruogu Fang, J. Daniel Fenn, Sue J. Goldie, Samuel M. Goldman, Paulina Gonzalez-Latapi, Diego L. Guarin, Brittany Kryzyzanowski, Nicole Lessard, Andi Lipstein Fristedt, Tejas R. Mehta, Monica Norcini, Jennifer L. Purks, Dawn Schiehser, Michael A. Schwarzschild, Caroline M. Tanner, Alexis Temkin, J. Bryan Unruh, Chris Vulpe, Kristin Wallock, Cordelia Walz, Allison W. Willis, Michael S. Okun

**Affiliations:** 1 Parkinson’s Foundation, New York, NY USA; 2 Duke University, Durham, NC USA; 3 Boston University, Boston, MA USA; 4 University of Florida, Gainesville, FL USA; 5 University of Alabama at Birmingham, Birmingham, AL USA; 6 Atria Health and Research Institute, New York, NY USA; 7 Harvard University, Cambridge, MA USA; 8 University of California at San Francisco, San Francisco, CA USA; 9 Northwestern University, Chicago, IL USA; 10 Barrow Neurological Institute, Phoenix, AZ USA; 11 Fresco Parkinson Institute Italia Onlus Foundation, Florence, Italy; 12 Massachusetts General Hospital, Boston, MA USA; 13 Environmental Working Group, Washington, DC USA; 14 University of Pennsylvania, Philadelphia, PA USA

**Keywords:** Diseases, Health care, Neurology, Neuroscience

## Abstract

This Perspective outlines the challenges addressed at the first Annual Parkinson’s Disease (PD) Prevention Think Tank, held on November 20–21, 2025 in Gainesville, Florida at the Fixel Institute. Over the course of two days, attendees from a wide variety of disciplines, including movement disorders neurology, environmental sciences, agriculture, public policy, public health, patient advocate groups, and more, discussed the current state of primary prevention research in PD, examined the lessons learned from prevention research in other disorders, and identified necessary regulatory and policy actions for moving toward primary prevention of PD. Eight key components of primary prevention are identified (8 C’s): accurately Count incidence and prevalence, Conduct longitudinal cohort studies, induce policy Change, Counsel on individual behavior change, Communicate clearly with the general public and with key stakeholders, form multi-centric Coalitions across neurodegenerative disorders, apply Cross-disciplinary approaches, and provide sustained financial Commitment to support ongoing prevention and mitigation efforts. These proceedings serve as an initial call-to-action for at-risk individuals, the clinicians who care for them, and for policymakers.

## Introduction

Parkinson’s disease (PD) is the world’s fastest-growing neurodegenerative disorder^1,2^. In 2025, an estimated 1.1 M individuals in North America were living with PD, with approximately 90,000 new diagnoses every year, making PD the second-most common neurodegenerative disorder after Alzheimer’s disease^3,4^. In light of an aging population and in the absence of a definitive disease-modifying treatment, the incidence and prevalence of PD are likely to grow rapidly in coming decades^2^, with profound health and economic impacts at the individual, family, health system, and societal levels^5–8^. Thus, strategies to prevent PD are urgently needed. Prevention may be subdivided into three types^9^: primary prevention to impede initiation of the disease process, secondary prevention to slow its progression in early or prodromal stages of disease prior to a traditional diagnosis, and tertiary prevention to minimize subsequent disability or disease-related complications. Studies of secondary^10^ and tertiary prevention^11^ in PD are underway, however primary prevention has received relatively little attention to date.

**Fig. 1:**
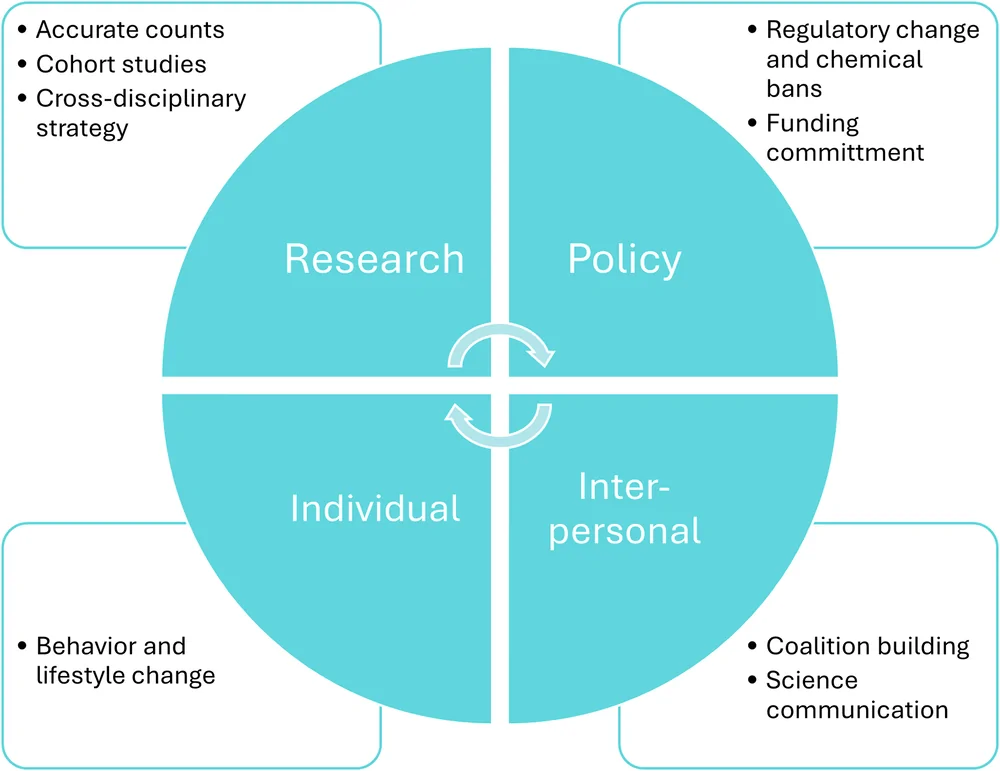
Multi-Pronged Approach and Calls-to-Action for Primary Prevention of Parkinson’s Disease. Primary prevention of a chronic, complex disorder like Parkinson’s disease requires a multi-pronged approach that interlaces research (accurate counts, cohort studies, and cross-disciplinary strategy), policy initiatives (regulatory change, chemical bans, and sustained funding commitments at federal and state levels), individual decisions (behavior and lifestyle changes), and interpersonal engagement (coalition building, science communication).

On November 20–21, 2025, the Parkinson’s Foundation and the Fixel Institute at the University of Florida convened a think tank to outline the current state of prevention research in PD, to examine the lessons learned from prevention research in other disorders, and to inform future regulatory and policy actions for primary prevention of PD. Over the course of two days, expert attendees from multiple healthcare, research, and policy disciplines (Table 1) discussed the urgent need for prevention and mitigation strategies, the impact of effective science communication, and the use of new technologies to track symptomatology at the individual and population levels. Consensus was reached on seven key action items (summarized as the 8 C’s) in Fig. 1.

**Table 1:** Think-Tank Attendees

Name	Institution/Affiliation
Rhoda Au, PhD	Boston University
Kelly Balmant, PhD	University of Florida
Anastasia Barnes, MD	University of Florida
Cynthia Barnett	University of Florida
James Beck, PhD	Parkinson’s Foundation
Kathleen Blake MD, MPH	Parkinson’s Foundation Patient Advisory Council
Bas Bloem MD, PhD, FRCPE	Radboud Medical Centre
Evangelos Christou, PhD	University of Florida
Wendy Dahl, PhD, RD	University of Florida
Laura Dalton	Harvard University
Briana de Miranda,PhD	University of Alabama at Birmingham
Alessandro Di Rocco, MD	Northwell Health
Ray Dorsey, MD, MBA	Atria Health and Research Institute
Ruogu Fang, PhD	University of Florida
J. Daniel Fenn MD, PhD	University of Florida
John Michael Gaziano MD, MPH	Massachusetts General Hospital/ Brigham
Sue Goldie MD, MPH	Harvard University
Samuel M. Goldman MD, MPH	University of California San Francisco
Paulina Gonzalez-Latapi, MD, Msc	Northwestern University
Diego Guarin, PhD	University of Florida
Kayln Henkel	Parkinson’s Foundation
Jonathan Kalmuss-Katz	EarthJustice
Jeongim Kim PhD	University of Florida
Brittany Krzyzanowski PhD	Barrow Neurological Institute
John Lehr	Parkinson’s Foundation
Nicole Lessard, BA, RN	Parkinson’s Foundation
Andi Lipstein Fristedt	Parkinson’s Foundation
Sneha Mantri MD, MS, FAAN	Parkinson’s Foundation
A. Enrique Martinez Nunez MD	University of Florida
Tejas R Mehta, MD	University of Florida
Monica Norcini PharmD, PhD	Fresco Parkinson Institute Italia Onlus Foundation
Michael Okun, MD	University of Florida Health, Norman Fixel Institute for Neurological Diseases
Peggy Borum, PhD	University of Florida
Jennifer L. Purks MD	University of Florida Health, Norman Fixel Institute for Neurological Diseases
Dawn Schiehser,PhD	University of Florida
Michael A. Schwarzschild MD, PhD	Massachusetts General Hospital
Karla Shelnutt PhD, RD	University of Florida, Institute of Food and Agricultural Sciences
Caroline (Carlie) Tanner, MD, PhD	University of California - San Francisco
Alexis Temkin, PhD	Environmental Working Group
J. Bryan Unruh, PhD	University of Florida, Institute of Food and Agricultural Sciences
Chris Vulpe MD, PhD	University of Florida, Center for Environmental and Human Toxicology
Kristin Wallock, OTD, MS, OTR/L	Parkinson’s Foundation
Cordelia Walz	Parkinson’s Foundation
Allison Willis MD, MS	University of Pennsylvania
Basma Yacoubi, PhD	University of Florida

## Current State of Prevention Research in PD

The Think Tank opened with a presentation on the geospatial analysis of PD in the United States. By overlaying individual patient data from the national Medicare dataset with land-use data from community use surveys, spatial and temporal trends in PD incidence, particularly in areas exposed to pesticides and chemicals, can be potentially uncovered^12^. For instance, agricultural land-use is strongly associated with PD prevalence, with populations in certain regions experiencing almost double the odds of PD^13^. This increased risk is modulated by additional geographic factors such as wind speed or groundwater sources^14^, as well as by chemical factors such as volatilization or environmental persistence. The data on specific chemical classes, including organochlorines^15^, organophosphates^16^, and chlorinated solvents^17^, were reviewed in detail. Notably, these risks are associations rather than definitive evidence of causation, reflecting the inherent challenges of causal inference in environmental epidemiology^18^. Observational studies are subject to potential confounding, exposure misclassification, and the complexity of long latency periods and mixed exposures across the life course. Because randomized exposure studies are not feasible, causal inference relies on integrating evidence across epidemiologic, toxicologic, and mechanistic studies, as well as frameworks such as the Bradford Hill criteria^19^. Approaches such as triangulating evidence from multiple data sources can strengthen causal interpretation while still acknowledging the remaining uncertainty.

From here, the conversation turned to mitigation principles. These were further divided into individual mitigation strategies, such as the use of personal protective equipment when applying pesticides^20^ and the protective effect of a healthy diet^21^, compared to societal mitigation strategies, such as the discontinuation or cleanup of toxic substances^22^. Exposure to chemical substances via different environmental media (e.g., water, soil, air) and routes of exposure (e.g., ingestion, topical, inhalation) and across the life-course were discussed. The Think Tank noted the paucity of data on real-world mixtures of different compounds, as well as exposure conditions in the host (e.g., age, sex, microbiome) that might prime certain individuals to develop disease. These effects may be mediated through biologically plausible mechanisms^23,24^, such as mitochondrial dysfunction, neuroinflammation, α-synuclein–related pathology, and related cellular stress pathways as well as through epigenetic mechanisms^25^. The neurotoxic potential of paraquat^26–28^, chlorpyrifos^29,30^, and trichloroethylene/perchloroethylene^17,31,32^, are well-documented in animal and epidemiological models of PD. Ongoing real-world research on chemical mixtures and routes of administration would further strengthen causal inference between exposure and disease, particularly by improving exposure characterization and reducing uncertainty in observed associations.

## Lessons Learned from Prevention Research in Other Disorders

Several analogies were made between PD and other disorders, including cancer, cardiac disease, and dementia. In each of these case examples, as in PD, the disease process begins years or decades before the onset of symptoms, providing both a challenge and an opportunity for prevention through improved screening processes, greater public awareness of individual risk, and more effective regulatory measures that can be employed to lower the risk of disease.

For instance, the link between cigarette smoke and lung cancer led to successful campaigns for smoking cessation (individual behavior change)^33,34^, smoking bans in many public areas, including schools, hospitals, and offices (organizational change), and the removal of some tobacco products from the market (regulatory change)^35^. These multi-pronged policy efforts ultimately led to a reduction in the incidence of lung cancer as well as a reduction in the risk of a second smoking-related condition, cardiovascular disease^36^. In contrast to current public awareness of the health risks of smoking, public awareness of PD remains relatively low^37^, suggesting a prime opportunity to improve effective science communication^38^ regarding both individual and community risk.

Considering the complexity of PD risk patterns, which are likely influenced by genetics as well as lifestyle, Think Tank participants emphasized the importance of multimodal data collection to enable triangulation of data points. For instance, genetic studies may be combined with fluid biomarkers, digital phenotypes, individual behaviors, and clinical correlates to provide a comprehensive understanding of the disease state and disease complexity. Specific examples from the Million Veteran Program^39^ and the Framingham Heart Study^40^ were discussed in detail. The possibility of using artificial intelligence and machine learning algorithms for early identification^41–44^, remote monitoring of disease progression^45^, and drug discovery^46,47^ were discussed as an important vehicle for multimodal data collection.

## Recommendations for Primary Prevention in PD

Think Tank participants reached consensus, leading to 8 key recommendations (8 C’s) to drive change in primary PD prevention (Fig. 1). These recommendations span all levels of the socioecological model of health promotion^48^ and bridge individual, interpersonal, research, and policy platforms.

### Counts of disease prevalence and incidence to examine secular trends

Epidemiological studies^1,2^ have revealed that both incidence and prevalence of PD are rising worldwide at a rate too high to be accounted for by the mere aging of the population or the impact of genetic factors alone. Our understanding of secular trends in PD epidemiology relies on consistent and accurate counts of disease. Administrative claims data, door-to-door community surveys, and clinical registries all have challenges and benefits. However, in the absence of accurate, precise, and consistent measures of disease burden, it will be impossible to determine the true impact of any prevention strategy. For instance, the creation of a PD registry, database, or cohort would allow insight into the impact of any given intervention. When policy changes are implemented in specific regions, it may be equally valuable to track incidence in surrounding or otherwise similar regions, which may serve as contemporary controls.

### Cohorts with deep phenotyping to integrate genetics, clinical, exposure, and digital data

The heterogeneity of PD may prove to be both an advantage and a challenge for prevention. It is likely PD has multiple upstream causes, and the interplay between them may prove challenging to untangle. Nevertheless, the need for well-phenotyped, diverse cohorts that integrate multiple data sources is clear. By combining genetic, clinical, digital, and exposure data, we will gain a greater understanding of gene-environment interactions and of the mechanistic complexity of disease processes, which may provide further insights into disease-modifying therapies.

### Changes in policy and regulation

Improved understanding of gene-environment interaction and disease mechanism can inform the policy and regulatory changes that are urgently needed to reduce exposure to neuro-toxicants. The U.S. Environmental Protection Agency (EPA) and other equivalent agencies around the world should take regulatory action to protect people from toxic chemicals linked to PD, such as paraquat, chlorpyrifos, trichloroethylene, and perchloroethylene. Where sufficient scientific data demonstrating these pesticides pose an unreasonable risk already exists, the EPA should initiate pesticide registration cancellation. And as new research about health and environmental exposure risks emerges, EPA should review evidence without delay to determine if the “reasonable certainty of no harm” standard has been violated or if the proposed uses lead to unreasonable risk. In the U.S., individual states can act to stop or stem exposure to chemicals; multiple states are already taking steps in this direction.

### Counseling around individual behavior change

Individual behavior changes, such as greater use of protective equipment, improved hygiene practices, and other strategies, could decrease an individual’s exposure risk to neurotoxic agents. Lifestyle changes, such as exercise and diet, are associated with a lower risk of PD^21,49–51^. Those with known genetic risks, strong family history of PD, or even prodromal features may be particularly motivated to make these changes. Given the long lead time between exposure and disease, however, some may not feel a pressing urgency to make lifestyle changes. In addition, given the broad set of factors that influence exposure (e.g., agricultural and industrial land-use patterns, occupational practices and worker protections, socioeconomic and environmental inequities) risk reduction will not be possible for many individuals. Finally, given the nature of environmental contamination of air, water, and soil, passive exposure to toxicants will remain a risk. This reality underscores the essential need for multi-layered intervention.

### Communication to educate the public and policymakers

Even though PD is the fastest-growing neurodegenerative condition worldwide and the second most common neurodegenerative condition in North America, public awareness of PD remains low^37^. Principles of effective science education and communication, such as clear and consistent messaging, tailored to audience needs, and infused with a call-to-action, should be adopted by researchers, clinicians, and policymakers.

### Coalition-formation across disease entities

The similarities between PD and other synucleinopathies, or PD and other neurodegenerative disorders, should be leveraged to achieve greater synergy. Changes at the individual, community, and national level that are beneficial for one disorder may have beneficial effects for another. Strong coalitions across multiple disease entities, such as the Neuro-Impact Coalition^52^ have the potential to attract attention that may reach beyond any individual disorder.

### Cross-disciplinary research and strategy

Think Tank attendees recognized that cross-disciplinary research and strategy need to be a top priority for PD prevention. To drive change on both an individual and a systems level, multiple stakeholder groups are needed. For instance, behavioral psychologists may provide greater insight into decision-making processes and tradeoffs for individuals. Health economists can provide data on the disease-related financial and non-financial burdens borne by families and communities. Molecular biologists and toxicologists can provide a mechanistic understanding of disease progression. The voices of people with PD were represented at the Think Tank and will continue to be critical to this work. As with other coalitions, a multi-disciplinary, multi-pronged approach is likely to yield the greatest benefit and impact for PD prevention.

### Commitment to fund research and mitigation

Research into PD prevention and mitigation will be critically important. Despite rising disease burden and rising cost of care, federally funded research in PD has remained flat since FY2021; when adjusted for inflation, this represents a decrease of approximately 20%. Disease-specific foundations are endeavoring to bridge the gap by providing additional funding for research and innovation, and some states are considering their own research investments. However, in the U.S., without a sustainable federal funding strategy, early-stage research may never reach the communities at greatest risk. As a starting point, the Parkinson’s community is advocating for $600 million dedicated for PD research by the NIH on an annual basis by 2028.

## Conclusion

Two hundred years after James Parkinson initially described PD, both global incidence and prevalence of the disease are rapidly rising. This rate of change suggests that environmental exposures and/or lifestyle factors may be contributing to the development of manifest motor PD, combined for some with an underlying genetic susceptibility. Such exposures may be active (e.g., spraying pesticides, ingesting dopamine-blocking or dopamine-toxic agents); others may be passive (e.g., volatilization of toxicants with distant spread, contamination of groundwater or other sources). Continued inaction will likely result in increasing rates of disability and premature death from PD, with profound economic consequences. We urge the Parkinson’s community in the U.S. and globally to consider the eight components (C’s) of primary prevention: accurate Counts of disease burden, longitudinal Cohort studies, policy Change, individual Counseling, science Communication, disease Coalitions, Cross-disciplinary approaches, sustained financial Commitment.

## Data Availability

No datasets were generated or analysed during the current study.
